# Effects of epigallocatechin-3-gallate on bovine oocytes matured *in vitro*

**DOI:** 10.5713/ajas.17.0880

**Published:** 2018-03-13

**Authors:** Ziqiang Huang, Yunwei Pang, Haisheng Hao, Weihua Du, Xueming Zhao, Huabin Zhu

**Affiliations:** 1 Embryo Biotechnology and Reproduction Laboratory, Institute of Animal Sciences, Chinese Academy of Agricultural Sciences, Beijing 100193, China

**Keywords:** Epigallocatechin-3-gallate, Reactive Oxygen Species (ROS), Oocytes, Oxidative Stress, Antioxidant, Bovine

## Abstract

**Objective:**

Epigallocatechin-3-gallate (EGCG) is a major ingredient of catechin polyphenols and is considered one of the most promising bioactive compounds in green tea because of its strong antioxidant properties. However, the protective role of EGCG in bovine oocyte *in vitro* maturation (IVM) has not been investigated. Therefore, we aimed to study the effects of EGCG on IVM of bovine oocytes.

**Methods:**

Bovine oocytes were treated with different concentrations of EGCG (0, 25, 50, 100, and 200 μM), and the nuclear and cytoplasmic maturation, cumulus cell expansion, intracellular reactive oxygen species (ROS) levels, total antioxidant capacity, the early apoptosis and the developmental competence of *in vitro* fertilized embryos were measured. The mRNA abundances of antioxidant genes (nuclear factor erythriod-2 related factor 2 [*NRF2*], superoxide dismutase 1 [*SOD1*], catalase [*CAT*], and glutathione peroxidase 4 [*GPX4*]) in matured bovine oocytes were also quantified.

**Results:**

Nuclear maturation which is characterized by first polar body extrusion, and cytoplasmic maturation characterized by peripheral and cortical distribution of cortical granules and homogeneous mitochondrial distribution were significantly improved in the 50 μM EGCG-treated group compared with the control group. Adding 50 μM EGCG to the maturation medium significantly increased the cumulus cell expansion index and upregulated the mRNA levels of cumulus cell expansion-related genes (hyaluronan synthase 2, tumor necrosis factor alpha induced protein 6, pentraxin 3, and prostaglandin 2). Both the intracellular ROS level and the early apoptotic rate of matured oocytes were significantly decreased in the 50 μM EGCG group, and the total antioxidant ability was markedly enhanced. Additionally, both the cleavage and blastocyst rates were significantly higher in the 50 μM EGCG-treated oocytes after *in vitro* fertilization than in the control oocytes. The mRNA abundance of *NRF2*, *SOD1*, *CAT*, and *GPX4* were significantly increased in the 50 μM EGCG-treated oocytes.

**Conclusion:**

In conclusion, 50 μM EGCG can improve the bovine oocyte maturation, and the protective role of EGCG may be correlated with its antioxidative property.

## INTRODUCTION

Reproduction is a complex process consisting of gametogenesis, fertilization, and embryonic and fetal development. Each event is so important that impairment of any of them may seriously affect subsequent embryonic development. Oocyte maturation is one such event that is crucial for fertilization and early embryonic development, and its disruption can potentially impair female reproduction [1]. However, the atmospheric oxygen concentration during *in vitro* maturation (IVM) results in significantly elevated reactive oxygen species (ROS) production, which is detrimental to subsequent embryonic development [2].

ROS is a collective term to describe oxygen radicals. Recent studies have shown that ROS are present at low concentrations in the female genital tract, which is beneficial for the sperm-oocyte fertilization process [3]. However, it is well known that excess ROS lead to oxidative stress, which damages DNA, lipids, and proteins, leading to defects and delays in embryonic development [4]. It has been reported that oxidative damage to lipids in the oocyte may result in persistently poor oocyte quality after early life exposure to several toxicants [5]. Studies have further demonstrated that oxidative stress interferes with oocyte maturation, which may influence early embryonic development, block two-cell embryos *in vitro* by modifying the key transcription factors, transforming gene expression and eventually resulting in female infertility [6].

Epigallocatechin-3-gallate (EGCG) is a major ingredient of catechin polyphenols in green tea and is considered to be one of the most bioactive chemical compounds due to its strong antioxidant properties [7]. EGCG has the ability to quench free radical species and chelate transition metals, which contributes to reducing oxidative stress levels [8]. It has been found that EGCG contributes to a reduced incidence of cancer [9]. Both *in vitro* and *in vivo* studies have suggested that EGCG can prevent Parkinson’s disease by decreasing oxidative stress and inflammatory reactions [10]. It has also been demonstrated that supplementing the culture medium with lower concentration of EGCG during *in vitro* fertilization (IVF) increases the fertilization rate, whereas higher EGCG concentrations in the culture medium tend to decrease the percentage of fertilized porcine oocytes [11].

Thus far, no studies have investigated the effect of EGCG on IVM of bovine oocytes. Thus, the aim of the present study was to investigate the effects of EGCG on IVM of bovine oocytes and on the cellular mechanisms that might be involved.

## MATERIALS AND METHODS

### Chemicals

Unless otherwise specified, all chemicals used in this study were purchased from Sigma-Aldrich Chemical Company (St. Louis, MO, USA).

### Oocyte collection and *in vitro* maturation

Bovine ovaries were collected from a local abattoir and transported to the laboratory in sterile physiological saline at 28°C to 30°C within 2 hours. The ovaries were washed thrice using sterile physiological saline at 37°C. Cumulus oocyte complexes (COCs) were aspirated from follicles ranging from 2 to 6 mm in diameter using an 18-gauge needle. Follicular fluid was collected in a 15-mL conical centrifuge tube and allowed to settle. COCs with oocytes surrounded by at least three layers of compact cumulus cells and uniform cytoplasm were selected for further experiments. Prior to IVM, the COCs were prewashed three times in IVM medium. Then, 50 COCs were evenly distributed in 750 μL of IVM medium covered with mineral oil in each well of a four-well plate (Nunclon, Roskilde, Denmark). Every time, a total of 100 COCs were cultured for each group. Finally, the COCs were incubated in humidified air with 5% CO_2_ at 38.5°C for 22 to 24 h. IVM was carried out in TCM-199 (Gibco, Grand Island, NY, USA) supplemented with 0.01 IU/mL follicle-stimulating hormone, 10 IU/mL luteinizing hormone, 1 μg/mL estradiol, and 10% (V/V) fetal bovine serum (FBS, Hyclone; Gibco BRL, Paisley, Scotland, UK).

### EGCG treatment

Technical grade EGCG was used to evaluate the effect of EGCG on oocyte maturation. EGCG was dissolved in pure water and diluted with IVM medium to gain a final concentration of 25, 50, 100, and 200 μM. The proportion of water in the final IVM medium should not exceed 1%.

### First polar body extrusion rate

To evaluate the effect of EGCG on nuclear maturation of mature oocytes, the first polar body extrusion rate was measured. After culture for 22 to 24 h, cumulus cells of mature COCs were removed by repeated gentle pipetting. The denuded oocytes were then observed under a microscope (Nikon Eclipse TE2000-s, Tokyo, Japan) to count the number of oocytes with the first polar body.

### Cortical granule staining

Cumulus cells of the matured COCs were removed by repeated gentle pipetting, and the zona pellucida of the bovine oocytes was removed by using 0.5% pronase in phosphate-buffered saline (PBS). The oocytes were fixed in PBS with 4% paraformaldehyde for 30 min, and then washed three times in sealing liquid (PBS with 0.3% bovine serum albumin [BSA] and 100 nM glycine). After all the samples were permeabilized in PBS supplemented with 0.1% Triton X-100 for 5 min, the oocytes were washed three times in sealing liquid for 5 min each time, and incubated in 20 μg/mL of fluorescein isothiocyanate (FITC) in the dark for 30 min at 37°C. The samples were washed three times in PBS-0.1% polyvinyl alcohol (PVA), and then observed on the slide with a cover glass under a confocal laser scanning microscope (Leica, TCS SP8, Wetzlar, Germany) with fluorescence excitation and emission at 492 nm and 518 nm, respectively.

### Mitochondrial activity staining

To study the effect of EGCG on the mitochondrial activity of *in vitro* matured oocytes, denuded oocytes were washed thrice in PBS-0.1% PVA. The oocytes were then incubated in MitoTracker Red CMXRos (Invitrogen, Carlsbad, CA, USA) in the dark for 20 min at 37°C. After the oocytes were incubated, they were washed three times in PBS containing 0.1% PVA. Oocytes were then fixed in PBS with 4% paraformaldehyde for 15 min. The fixed oocytes were washed three times in PBS-0.1% PVA. Finally, the oocytes were observed on a slide with a cover glass under a confocal laser scanning microscope (Leica, TCS SP8, Germany) with the fluorescence excitation and emission at 579 nm and 599 nm, respectively.

### Cumulus cells expansion

Cumulus expansion index (CEI) was scored after 22 h of IVM according to the scoring system of Vanderhyden et al [12]. Briefly, a score of 0 indicates no detectable response; score 1 indicates no cumulus cell expansion but cells appear as spherical; score 2 indicates only the outermost layers of cumulus cells expanded; score 3 indicates all cell layers have expanded except the corona radiata; and score 4 indicates expansion has occurred in all cell layers including the corona radiate.

The abundance of cumulus expansion-related genes is another aspect to evaluate oocyte maturation. The expansion of cumulus cells was further determined by determining the levels of cumulus expansion-related genes, including hyaluronan synthase 2 (*HAS2*), tumor necrosis factor alpha induced protein 6 (*TNFAIP6*), pentraxin 3 (*PTX3*), and prostaglandin (*PTGS2*).

Cumulus cells were collected from COCs matured *in vitro*, washed three times in PBS, and stored in liquid nitrogen until RNA extraction. Total RNA was extracted from the cumulus cells of 100 COCs in each group using Trizol reagent (Invitrogen, USA), and quantified by measuring the absorbance at 260 nm. First-strand cDNA was synthesized using 0.05 μg of RNA with random hexamers. The levels of cumulus cell expansion-related genes, including *HAS2*, *TNFAIP6*, *PTX3*, and *PTGS2*, were determined by quantitative real-time polymerase chain reaction (RT-PCR) in three replicates using a BIO-RAD CFX96 (BIO-RAD, Hercules, CA, USA) instrument. Table 1 lists the primers used for the analysis.

Reverse transcription was conducted using a RevertAid First-Strand cDNA Synthesis Kit (Thermo Fisher Scientific, Waltham, MA, USA). Briefly, a total reaction volume of 12 μL including 0.3 μg total RNA, 1 μL of oligo(dT) 18 primer, and nuclease-free water was mixed in a nuclease-free microfuge tube, heated to 65°C for 5 min. Then, 4 μL of 5× reaction buffer, 1 μL of RiboLock RNAse Inhibitor (20 U/μL), 2 μL of 10 mM dNTP mix, and 1 μL of RevertAid M-MuLV RT (200 U/μL) were added to this reaction mixture, which was then incubated at 42°C for 60 min, followed by 70°C for 5 min. The resultant cDNA was stored at −20°C until use.

The RT-PCR amplification mixture consisted of 2 μL cDNA, 12.5 μL of 2× SYBR Premix Ex Taq (Takara, Tokyo, Japan), and 0.5 μL each of forward and reverse primers (Table 1) in a total volume of 25 μL. The PCR program was as follows: 95°C for 30 s; 39 cycles of 95°C for 5 s, annealing at 60°C for 30 s, a melting curve from 95°C for 10 s, 65°C for 5 s, and 95°C for 5 s, followed by a final cooling step at 4°C. The expression levels of cumulus cell expansion-related genes were calculated using glyceraldehyde phosphate dehydrogenase (*GAPDH*) as a reference gene.

### ROS level detection

To explore the effect of EGCG on the ROS level of *in vitro* matured oocytes, the cumulus cells of COCs were removed by repeated gentle pipetting. The denuded oocytes were then washed three times in PBS containing 0.1% PVA. The oocytes were incubated for 20 min in the dark in 50 μM 2′,7′-dichlorofluorescein diacetate (H_2_DCFDA) at 37°C. The oocytes were then washed three times in PBS-0.1% PVA. Finally, the oocytes were observed under a fluorescence microscope (Nikon, Japan). The fluorescence intensities of oocytes were analyzed by Image J software (Version 1.49v, National Institute of Health, Bethesda, MD, USA) and normalized to that of the control oocytes.

### Detection of oocyte apoptosis

To detect the externalization of phosphatidylserine in early apoptotic bovine oocytes, Annexin V –FITC staining was performed according to the manufacturer’s instruction (Life Technologies Inc., Grand Island, NY, USA). Briefly, 20 to 30 matured bovine oocytes were first washed thrice in PBS-0.1% PVA, after which the oocytes were washed with 1× annexin-binding buffer for 5 min, then the oocytes were incubated in 490 μL 1× annexin-binding buffer supplemented with 10 μL Annexin V conjugate for 20 min at room temperature. Finally, oocytes were washed again as above, deposited onto slide and detected under a laser-scanning confocal microscope (Leica, TCS SP8, Germany).

### Total antioxidant capability measurement

The total antioxidant capability (T-AOC) levels of mature oocytes were measured using the total antioxidant capability measurement kit (Jiancheng, Nanjing, China) according to the manufacturer’s instructions. Briefly, 100 oocytes in different treatment groups were homogenized in 100 μL PBS and sonicated at 50 W for 1 min, then centrifuged at 3,000 g for 5 min at 4°C. The cellular supernatant was added to ABTS (2,2′-azino-bis[3-ethylbenzthiazoline-6-sulfonic acid]) reagent in a microplate and incubated at room temperature for 6 min. The absorbance was measured in a microplate reader (SpectraMax M5, Molecular Devices, Sunnyvale, CA, USA) at 414 nm.

### Expression of oxidative stress-related genes

*In vitro* matured oocytes were collected, washed three times in PBS-0.1% PVA, and stored in the liquid nitrogen until RNA extraction. One hundred oocytes were obtained from each group for RT-PCR. The process was same as the measurement of cumulus cell expansion-related genes. We then determined the expression levels of the oxidative stress-related genes nuclear factor erythriod-2 related factor 2 (*NRF2*), superoxide dismutase 1 (*SOD1*), catalase (*CAT*), and glutathione peroxidase 4 (*GPX4*) using *GAPDH* as a reference gene. Table 1 lists the primers used for the analysis.

### *In vitro* fertilization

The IVF was conducted following the method described by Pang et al [13]. Mature bovine oocytes were washed thrice in Brackett and Oliphant (BO) fertilization medium containing 10 μg/mL heparin and 4 mg/mL fatty acid-free BSA. The surrounding cumulus cells were partially denuded by gentle pipetting, and 15 to 20 COCs were then transferred into a 50-μL drop of fertilization medium. Frozen semen was thawed in a water bath at 37°C for 30 s, then the sperm was washed twice in 6 mL BO wash medium by centrifugation at 615 g for 5 min. The concentration of the sperm was determined after the final wash, then a 50-μL aliquot of sperm suspension with a concentration of 10×10^6^ spermatozoa/mL was added to each fertilization drop, and the mixture was incubated for 8 to 18 h at 38.5°C in 5% CO_2_ in air with high humidity. The cumulus cells of presumptive zygotes and adherent spermatozoa were stripped by gentle pipetting. Then, 25 to 30 presumed zygotes were cultured for 48 h in 100-μL droplets of CRlaa medium containing 6 mg/mL of fatty acid-free BSA. After 48 h, cleaved embryos were chosen for further culture in CRlaa medium containing 10% (V/V) FBS for another 5 days. Half of the medium was removed and replaced with fresh medium every 48 h. The cleavage and blastocyst rates were determined on days 2 and 7, respectively.

### Statistical analysis

All the experiments were conducted at least three times. The results were expressed as means±standard error of the mean. Percentage data were subjected to arcsine transformation before statistical analysis. One-way analysis of variance was used for data analysis, followed by multiple comparisons using Statistical Analysis System software (SAS Institute, Cary, NC, USA). The relative mRNA expression levels were expressed as fold change (2^−ΔΔCT^), and data were analyzed by the Kruskal-Wallis test followed by pairwise comparisons using IBM SPSS Statistics 22.0. Differences with p<0.05 were considered significant.

## RESULTS

### Effects of EGCG on the first polar body extrusion rate

To explore the effect of EGCG on the nuclear maturation of bovine oocytes, the first polar body of mature oocytes was observed. Exposure to 25, 50, and 100 μM EGCG improved the first polar body extrusion rates, while 200 μM EGCG suppressed the extrusion rate. The first polar body extrusion rate was significantly higher in the 50 μM EGCG (88.04%± 2.49%) group compared to the control group (76.04%±1.22%, p<0.05; Table 2). This indicated that 50 μM EGCG played an important role in enhancing the nuclear maturation of bovine oocytes.

### Effect of EGCG on the distribution of cortical granules

**Figure 1:**
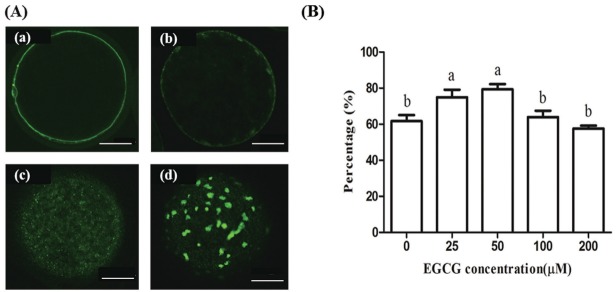
Effect of epigallocatechin-3-gallate (EGCG) on the distribution of cortical granules in mature bovine oocytes. (A) Representative photomicrographs: (a) peripheral distribution; (b) cortical distribution; (c) homogeneous distribution; (d) cluster distribution. (B) Percentage of oocytes with peripheral and cortical distribution of cortical granules in each treatment group. Scale bar, 25 μm. Superscript letters (^a,b^) indicate significant differences (p<0.05).

Cortical granule staining was used to evaluate the cytoplasmic maturation of bovine oocytes. There were significant differences in the percentage of oocytes with cortical granules in the peripheral and cortical regions between the 25 and 50 μM EGCG groups compared to the control group (p<0.05); but there was no significant difference of other tested groups compared to the control group (Figure 1).

### Effect of EGCG on mitochondrial activity

**Figure 2:**
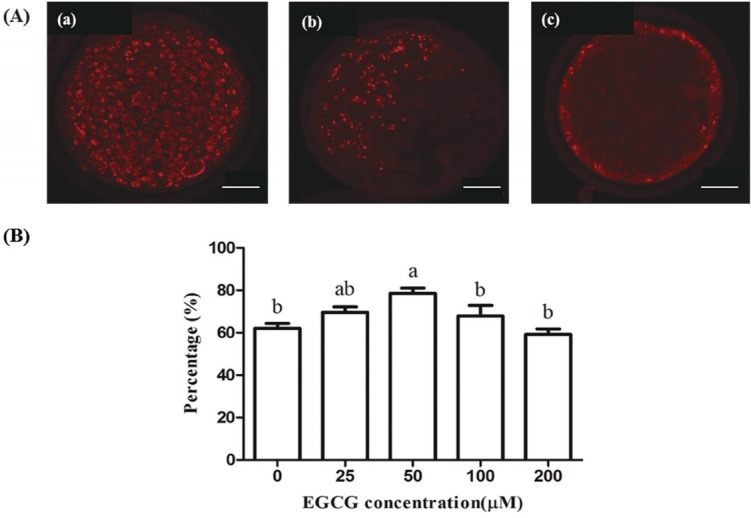
Effect of epigallocatechin-3-gallate (EGCG) on the distribution of mitochondria in bovine oocytes. (A) Representative photomicrographs: (a) homogeneous distribution; (b) semi-peripheral distribution; (c) peripheral distribution. (B) Percentage of oocytes with homogeneous distribution of mitochondria in each treatment group. Scale bar, 25 μm. Superscript letters (^a,b^) indicate significant differences (p<0.05).

Mitochondrial distribution in oocytes is another indicator of oocyte maturation. We therefore determined the proportions of bovine oocytes with homogeneous distribution of mitochondria in each group. The proportion of bovine oocytes with homogeneous mitochondrial distribution were improved in the 25, 50, and 100 μM EGCG groups, and it was significantly higher in the 50 μM EGCG group compared to the control group (p<0.05), but there was no statistic difference among other groups (Figure 2).

### Effect of EGCG on the cumulus cells expansion

**Figure 3:**
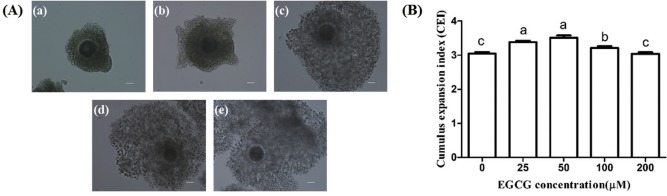
Effects of epigallocatechin-3-gallate (EGCG) on the cumulus expansion index of bovine cumulus-oocytes complexes. (A) Representative photomicrographs: (a) score 0, no expansion; (b) score 1, no cumulus cell expansion but cells appear as spherical; (c) score 2, only the outermost layers of cumulus cells have expanded; (d) score 3, all cell layers have expanded except the corona radiata; (e) score 4, expansion has occurred in all cell layers including the corona radiata. (B) Cumulus expansion index in different EGCG treatment groups. Scale bar, 50 μm. Superscript letters (^a,b,c^) indicate significant differences (p<0.05).

As shown in Figure 3, the CEI of 25, 50, and 100 μM EGCG groups were significantly higher than that of the control group (p<0.05), but there was no significant differences between 200 μM EGCG group and the control group.

**Figure 4:**
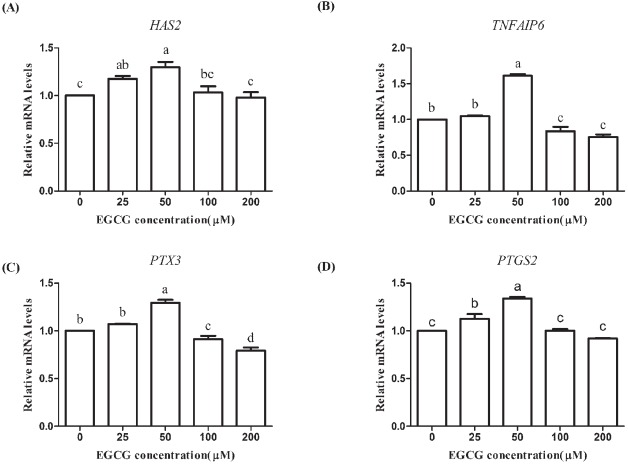
Effects of epigallocatechin-3-gallate (EGCG) on the mRNA abundance of cumulus expansion-related genes in mature bovine cumulus-oocyte complexes. (A) hyaluronan synthase 2 (*HAS2*); (B) tumor necrosis factor alpha induced protein 6 (*TNFAIP6*); (C) pentraxin 3 (*PTX3*); (D) prostaglandin 2 (*PTGS2*). Superscript letters (^a,b,c,d^) indicate significant differences (p<0.05).

The expression levels of cumulus expansion-related genes (*HAS2*, *TNFAIP6*, *PTX3*, and *PTGS2*) were increased in the 25 and 50 μM EGCG groups but decreased in the 200 μM EGCG group and the expression levels in the 50 μM EGCG group were significantly higher compared to the corresponding levels in the control group (p<0.05; Figure 4). For 100 μM EGCG group, the mRNA expression levels of *HAS2* and *PTGS2* were significantly higher (p<0.05; Figure 4), but the levels of *TNFAIP6* and *PTX3* were lower compared with the control group. These results demonstrated that 50 μM EGCG contributed to the expansion of cumulus cells of COCs.

### Effect of EGCG on the ROS levels

**Figure 5:**
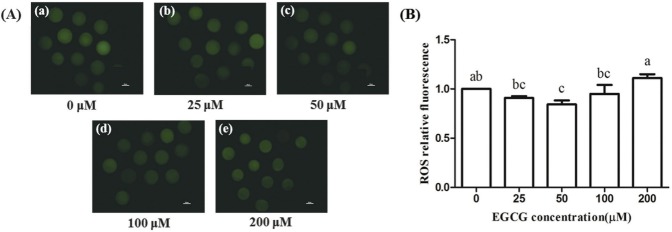
Effect of epigallocatechin-3-gallate (EGCG) on the relative fluorescence of reactive oxygen species (ROS) in bovine oocytes. (A) Representative photomicrographs in different EGCG treatment groups. (B) ROS relative fluorescence in each EGCG treatment group. Scale bar, 50 μm. Superscript letters (^a,b,c^) indicate significant differences (p<0.05).

The ROS levels of bovine oocytes were lower in the 25, 50, and 100 μM EGCG groups than in the control group, and this difference was statistically significant in the 50 μM group (p< 0.05), but no statistic differences were observed between other tested groups and the control group (Figure 5).

### Effect of EGCG on the early apoptosis of matured bovine oocytes

**Figure 6:**
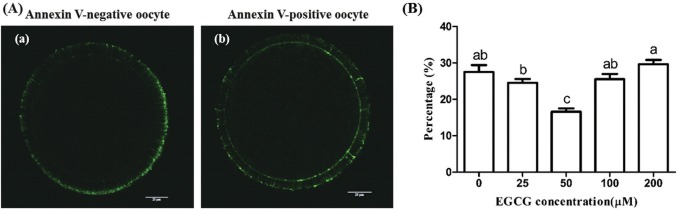
Effect of epigallocatechin-3-gallate (EGCG) on the early apoptosis of mature bovine oocytes. (A) Representative photomicrographs: (a) Annexin V-negative oocyte which only has fluorescent signals on the zone; (b) Annexin V-positive oocyte which has fluorescent signals on both the zona and the membrane. (B) Percentage of the early apoptosis bovine oocytes in different EGCG treatment groups. Scale bar, 25 μm. Superscript letters (^a,b,c^) indicate significant differences (p<0.05).

Annexin-V assay showed that the early apoptotic rate in bovine oocytes exposed to 50 μM EGCG (16.62%±3.29%) was significantly lower than that in the control group (27.52%± 1.59%) (p<0.05). While no statistically significant differences were observed between other tested groups and the control group (Figure 6).

### Effect of EGCG on the total antioxidant capability

**Figure 7:**
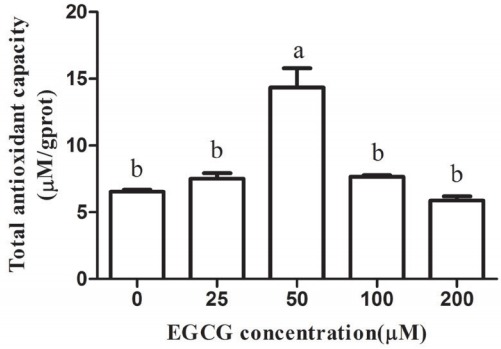
The total antioxidant capacity of mature bovine oocytes in each epigallocatechin-3-gallate (EGCG) treatment group. Superscript letters (^a,b^) indicate significant differences (p<0.05).

As expected, the T-AOC was higher in the 25, 50, and 100 μM EGCG groups compared with the control group. Compared to the control group, the T-AOC level was significantly higher in the 50 μM group (p<0.05), but no statistically significant differences were observed between other tested groups and the control group (Figure 7).

### Effects of EGCG on the expression of oxidative stress-related genes

**Figure 8:**
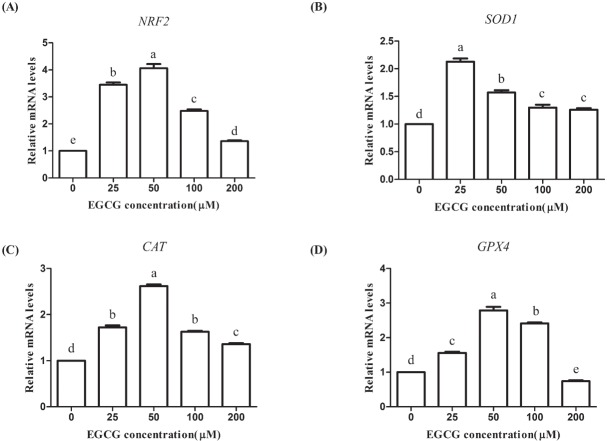
Effect of epigallocatechin-3-gallate (EGCG) on mRNA abundances of oxidative stress-related genes in bovine oocytes. (A) nuclear factor erythriod-2 related factor 2 (*NRF2*); (B) superoxide dismutase 1 (*SOD1*); (C) catalase (*CAT*); (D) glutathione peroxidase 4 (*GPX4*). Superscript letters (^a,b,c,d^) indicate significant differences (p<0.05).

We used RT-PCR to determine the relative mRNA abundance of genes related to oxidative stress (*NRF2*, *SOD1*, *CAT*, and *GPX4*). The mRNA levels of the *NRF2*, *SOD1*, and *CAT* genes were significantly higher in all the EGCG groups than those in the control group (p<0.05). The *GPX4* level was significantly improved in the 25, 50, and 100 μM groups but markedly reduced in the 200 μM group (p<0.05; Figure 8).

### Effect of EGCG pretreatment of bovine oocytes on the developmental competence of *in vitro* fertilized embryos

EGCG improved the cleavage rates of bovine oocytes at all concentrations except 200 μM (75.48%±1.79%) compared to the control group (76.07%±3.34%), and 50 μM EGCG pretreatment (86.23%±2.90%) resulted in a significant increase in cleavage rate (p<0.05). No statistically significant difference was observed between the 25 μM (80.74%±5.74%) and 100 μM (80.33%±0.92%) treatment group. The blastocyst rates were significantly higher in the 25, 50, and 100 μM groups (30.28%±1.83%, 36.34%±0.62%, and 28.68%±3.68%, respectively) compared to the control group (22.42%±1.83%; p<0.05), but the blastocyst rate in the 200 μM group (18.30%±0.12%) was significantly lower than that in the control group (p<0.05; Table 3).

## DISCUSSION

It is well known that ROS are both beneficial and detrimental in terms of important regulatory functions on spermatozoa [14] and oocytes [15]. Excessive ROS accumulation results in oxidative stress, which may negatively impact oocyte physiology by inducing apoptosis [16]. Recently, green tea (GT) supplementation has been reported to possess characteristics that may enhance the quality of male and female gametes largely due to the ability of catechin polyphenols to quench ROS [7]. Wang et al [17] showed that the presence of 15 μM GT polyphenols (GTP) during IVM culture efficiently improved fertilization and development competence of bovine oocytes, and this improvement was correlated with increase of intracellular glutathione concentration after IVM of oocytes.

EGCG is considered the most abundant catechin in GT infusions and one of the most promising bioactive compounds known for its strong antioxidant properties [7]. In the present study, we showed that EGCG can benefit bovine oocyte maturation *in vitro*. In particular, adding 50 μM EGCG to IVM media resulted in significant improvement in cumulus expansion, nuclear and cytoplasmic maturation, as well as subsequent embryonic development. A previous study provided evidence that *in vivo* administration of the antioxidant EGCG can alleviate the negative effects of hyperthermia on developmental competence of the ovarian pool of oocytes, as reflected by an increased rate of early cleaved embryos and blastocyst formation [18]. Spinaci et al [11] also demonstrated that supplementation of EGCG at a lower concentration (10 μg/mL) during IVF significantly increased the fertilization rate, while higher EGCG concentrations (25 μg/mL) decreased the percentage of fertilized oocytes in porcine. Another study reported that high doses of EGCG in IVM were harmful to the oocytes as evidenced by the decreased quality of embryos derived from somatic cell nuclear transfer, and EGCG had no beneficial effects on *in vitro* development of pig cloned embryos. However, the intracellular ROS level was significantly lower in the parthenogenetic activation embryos cultured in 10 μg/mL EGCG than the other groups [19]. The improvement in oocyte maturation potential observed in this study might be due to the antioxidant effect of EGCG, as supplementation of IVM medium with 50 μM EGCG not only decreased the intracellular ROS level and the early apoptotic rate, but also enhanced the T-AOC of mature bovine oocytes, which underlaid the beneficial effect noted later during development in the treated group.

The decreased intracellular ROS level upon EGCG treatment may contribute to its direct radical scavenging activity or to the EGCG-induced increase of antioxidative enzyme activity. In the present study, it was found that the mRNA levels of *SOD1*, *CAT*, and *GPX4* were significantly upregulated in EGCG treatment groups. SOD is one part of the enzyme defense system against oxidative stress by converting superoxide radical anion to H_2_O_2_. CAT, which is expressed in the majority of the cells, organs, and tissues, represents the enzyme involved in the reductive depletion of H_2_O_2_ to water. GPX is a selenium-containing enzyme, which catalyses both the reduction of H_2_O_2_, and organic hydroperoxides to water or corresponding alcohols [20]. Meng et al [21] reported that human diploid fibroblasts (HDF) treated with EGCG at 25 and 50 μM for 24 h considerably increased *CAT*, *SOD1*, *SOD2*, and glutathione peroxidase (*GSH-Px*) gene expression and their enzyme activities, thus protecting HDF against H_2_O_2_-induced oxidative damage. Supplemental EGCG decreased hepatic malondialdehyde level, and increased hepatic SOD, CAT, and GSH-Px activities in heat-stressed quails [22]. A recently published study which focused on the protective effects of EGCG in preventing arsenic-induced hepatotoxicity showed that arsenic decreased the antioxidant enzymes SOD, GPX, and CAT activities and the decrease was significantly inhibited by treatment of EGCG [23]. Our results were consistent with the above, and indicate that the antioxidative effect of EGCG in bovine oocyte may be largely attributed to its modulation of the enzymatic defense system, namely SOD1, CAT, and GPX4.

Following ROS exposure, the organism could form a complex oxidative stress response system by inducing the production of a series of protective proteins to alleviate cellular damage. This coordinated response is regulated by the antioxidant response element (ARE) of the protective genes upstream of the regulatory region. NRF2 is not only an activator of ARE but also a receptor of exogenous toxic substances and oxidative stress. It plays a rather important role in alleviating oxidative damages. It has been reported that NRF2 plays a crucial role in regulating mitochondrial redox homeostasis and supporting both the structural and functional integrity of mitochondria, especially under conditions of oxidative stress [24]. Sriram et al [25] investigated the mechanism involved in the enhancement of antioxidant activities after EGCG treatment during bleomycin-induced pulmonary fibrosis and found that the beneficial effect of EGCG against lung injury was associated with the Nrf2-based activation of the oxidative stress response. EGCG attenuated the retention of arsenic in liver tissues and improved the expression of Nrf2 signaling related genes (Nrf2, Nqo1, and Ho-1) [23]. In this study, enriching the maturation media with EGCG significantly elevated the transcript level of *NRF2*. Our results suggest that EGCG might alleviate oxidative stress in oocytes during IVM by activating Nrf2 pathway. However, additional details will be required to ascertain the exact mechanism involved in this process.

In conclusion, the results of the present study show that treatment with 50 μM EGCG during IVM culture efficiently improves both nuclear and cytoplasmic maturation of bovine oocytes and subsequent developmental competence of embryos. The protective effect of EGCG in this process may be partly due to its antioxidative property.

## Figures and Tables

**Table 1:** Primer sequences for cumulus cell expansion-related and oxidative stress-related genes

Gene	Primer sequences	Product size (bp)	Accession No.
*GAPDH*	5′-GGGTCATCATCTCTGCACCT 5′-GGTCATAAGTCCCTCCACGA	176	NM_001034034.2
*HAS2*	5′-GGATCTCCTTCCTCAGCAGTGT 5′-ATTCCCAGAGGTCCGCTAATG	106	NM_174079.2
*TNFAIP6*	5′-TGAAAGATGGGATGCATATTGC 5′-CATTTGGGAAGCCTGGAGATT	101	NM_001007813.2
*PTX3*	5′-CATGTATGTGAATTTGGACAACGA 5′-GCTTGTCCCACTCGGAGTTC	101	NM_001076259.2
*PTGS2*	5′-CTTAAACAAGAGCATCCAGAATGG 5′-GCTGTACGTAGTCTTCAATCACAATCT	106	NM_174445.2
*NRF2*	5′-GATGGACTTGGAGCTGCCG 5′-GCTCATGCTCCTTCTGTCGT	140	NM_001011678.2
*SOD1*	5′-CCACGTCCATCAGTTTGGAGA 5′-CTTTTGGCCCACCGTGTTTT	92	NM_174615.2
*CAT*	5′-CTGGGACCCAACTATCTCCA 5′-GATGCTCGGGAGCACTAAAG	148	NM_001035386.2
*GPX4*	5′-GTGCTCGCTCCATGCACGA 5′-CCTGGCTCCTGCCTCCCA	223	NM_001034034.2

*GAPDH*, glyceraldehyde phosphate dehydrogenase; *HAS2*, hyaluronan synthase 2; *TNFAIP6*, tumor necrosis factor alpha induced protein 6; *PTX3*, pentraxin 3; *PTGS2*, prostaglandin 2; *NRF2*, nuclear factor erythriod-2 related factor 2; *SOD1*, superoxide dismutase 1; *CAT*, catalase; *GPX4*, glutathione peroxidase 4.

**Table 2:** Effect of EGCG on the first polar body extrusion rate of bovine oocytes

Concentration of EGCG (μM)	NO. of oocytes	NO. of oocytes extruding the first polar body	The first polar body extrusion rate (%±SE)
0	217	165	76.04±1.22^bc^
25	156	125	80.17±0.76^b^
50	204	180	88.04±2.49^a^
100	162	125	76.95±4.51^bc^
200	163	116	71.57±5.21^c^

EGCG, epigallocatechin-3-gallate; SE, standard error.

Mean values within the same column with different superscript letters (^a,b,c^) differ significantly (p<0.05).

**Table 3:** Effect of EGCG in the maturation medium on the developmental competence of bovine oocytes

Concentration of EGCG (μM)	No. of oocytes	Cleavage rate (%±SE)	Blastocyst rate (%±SE)
0	109	76.07±3.34^b^	22.42±1.83^c^
25	115	80.74±5.74^ab^	30.28±2.15^b^
50	137	86.23±2.90^a^	36.34±0.62^a^
100	113	80.33±0.92^ab^	28.68±3.68^b^
200	116	75.48±1.79^b^	18.30±0.12^d^

EGCG, epigallocatechin-3-gallate; SE, standard error.

Mean values within the same column with different superscript letters (^a,b,c,d^) differ significantly (p<0.05).
